# Effects of experimentally induced cervical spine mobility alteration on the postural organisation of gait initiation

**DOI:** 10.1038/s41598-022-10101-6

**Published:** 2022-04-11

**Authors:** A. Delafontaine, T. Vialleron, D. G. Diakhaté, P. Fourcade, E. Yiou

**Affiliations:** 1 CIAMS, Université Paris-Saclay, 91405 Orsay, France; 2 CIAMS, Université d’Orléans, 45067 Orléans, France; 3 Gaston Berger University, Saint-Louis UFR Sciences de l’Education de la Formation et du Sport, Saint Louis, Senegal; 4 Laboratory CIAMS, Université Paris-Saclay, Rue Pierre de Coubertin, 91405 Orsay Cedex, France

**Keywords:** Motor control, Biomedical engineering

## Abstract

Gait initiation (GI), the transient period between quiet standing and locomotion, is a functional task classically used in the literature to investigate postural control. This study aimed to investigate the influence of an experimentally-induced alteration of cervical spine mobility (CSM) on GI postural organisation. Fifteen healthy young adults initiated gait on a force-plate in (1) two test conditions, where participants wore a neck orthosis that passively simulated low and high levels of CSM alteration; (2) one control condition, where participants wore no orthosis; and (3) one placebo condition, where participants wore a cervical bandage that did not limit CSM. Centre-of-pressure and centre-of-mass kinematics were computed based on force-plate recordings according to Newton’s second law. Main results showed that anticipatory postural adjustments amplitude (peak backward centre-of-pressure shift and forward centre-of-mass velocity at toe-off) and motor performance (step length and forward centre-of-mass velocity at foot-contact) were altered under the condition of high CSM restriction. These effects of CSM restriction may reflect the implementation of a more cautious strategy directed to attenuate head-in-space destabilisation and ease postural control. It follows that clinicians should be aware that the prescription of a rigid neck orthosis to posturo-deficient patients could exacerbate pre-existing GI deficits.

## Introduction

According to the biomechanical concept of “posturo-kinetics capacity”, movement induces a perturbation to posture and balance, and a counter-perturbation has to be developed to limit the perturbation effects, which is a condition necessary to perform the movement efficiently^1,2^. The counter-perturbation is produced in the body segments that constitute the “postural chain” and is known to be triggered before the onset of the perturbation, i.e. during so-called “Anticipatory Postural Adjustments” (APA^3–6^). According to the posturo-kinetics capacity concept, the efficiency of these APA depends on the mobility of the postural chain, corresponding to the range of motion of the postural segments involved in APA. Any factor that limits this mobility would have a negative impact on APA development, which, in turn, would result in decreased motor performance and/or stability. This concept has received a large amount of experimental support in the last decades, with studies investigating the effect of a restriction in the mobility of different parts of the postural chain on the postural organisation of various segmental or whole body movements, such as isometric ramp push^7^, arm pointing to a target^8^, trunk flexion^9^, bilateral forward arm reach^10^, sit-to-stand^9^, and gait initiation^11^. In these studies, mobility of the postural chain was manipulated by means of orthoses of different rigidities at the ankle^12^, knee^11^ or lower back^13^, by changing the initial postural conditions^7^, with ankle stretching^14^ or with spinal manipulation^15^.

To date, the effect of alteration of cervical spine mobility on the postural organisation of a whole-body movement has been investigated in only one study^16^. These authors reported that, in healthy young adults performing series of sit-to-stand tasks, the experimental restriction of cervical spine mobility induced by the wearing of cervical orthoses of different rigidities (from low to high), resulted in a reorganisation of APA and lower motor performance. However, these authors did not investigate postural stability and restricted their analysis solely to centre-of-pressure data. Therefore, the influence of cervical spine mobility restriction on the postural organisation of a locomotor task involving 3D whole-body centre-of-mass motion and asymmetrical leg displacements remains to be clarified.

It is noteworthy that alteration of cervical spine mobility also occurs with physiological aging^17,18^ and in many pathologies such as cervical spondylosis, chronic neck pain, whiplash injury etc. It may also occur in patients with Parkinson’s disease (PD) due to increased neck tone^19^. Of particular interest, Franzén et al. (2009) reported that increased neck tone in PD was associated with reduced performance in balance, walking and turning tasks^20^. These results, along with those of Hamaoui and Alamini-Rodrigues in healthy young adults, are consistent with the assumption that the alteration of cervical spine mobility with aging or, to a greater extent, with PD, might contribute to the gait and balance disorders classically reported in these populations. However, it is clear that the causes of these motor disorders are multifactorial and cannot be ascribed solely to this alteration alone. One way to get further insight into the effect of cervical spine mobility alteration on the postural organisation of a locomotor task is to have healthy participants initiate gait while wearing cervical spine orthoses of different rigidities.

Gait initiation is a functional locomotor task that corresponds to the transition phase from the upright standing posture to swing foot-contact with the support surface^21,22^. It is composed of a postural phase preceding swing foot-off (corresponding to the APA phase), followed by an execution phase ending at foot-contact. During APA, the centre-of-pressure is shifted backward toward the heels and laterally toward the swing leg side. The backward centre-of-pressure shift provides the initial (toe-off) propulsive forces necessary to reach the intended forward centre-of-mass velocity/step length at the end of gait initiation (corresponding to motor performance^23^), while the mediolateral centre-of-pressure shift serves to propel the centre-of-mass toward the forthcoming stance foot so as to maintain postural stability during the execution phase^22,24^. The backward centre-of-pressure shift is known to be induced by bilateral soleus inhibition followed by strong bilateral tibialis anterior activation^25^, while the mediolateral centre-of-pressure shift is induced by complex synergy involving the musculature of the hips, knees and ankles^22^. In addition, during the mid-execution phase, the centre-of-mass falls under the gravity effect, as shown by an increasing downward centre-of-mass velocity. This centre-of-mass fall is actively braked before swing foot-contact, as shown by a reversal of the vertical centre-of-mass velocity trace. This active braking, which is caused by stance leg triceps surae activation, is thought to reflect balance control during gait initiation^26–28^.

These studies on gait initiation show that the anticipatory centre-of-pressure shift and the active centre-of-mass brake do not directly involve cervical spine mobility, but merely involve ankle, knee and hip mobility. As stated above, constraining mobility at these joints with an orthosis induces APA reorganisation and an alteration of performance and/or stability. Now, it may be stressed that during locomotion, trunk accelerations might potentially destabilise the head-in-space position^17^. However, head-in-space position has been shown to remain highly stable during locomotor tasks such as gait initiation^17^, free walking^29^, walking in place, running in place and hopping^30–33^. Based on this evidence, it has been proposed that the head, which contains the visual and vestibular systems, forms a frame of reference for the perception of movement, control of limb coordination and body balance^32,34^. This stabilisation of the head during locomotion has been shown to involve compensatory rotations of the head with respect to trunk translational motion^17,32,35–38^, i.e. cervical spine mobility. It follows that constraining cervical spine mobility by making it more difficult to control head stability, may be expected to result in the alteration of the postural control mechanisms involved in gait initiation.

The aim of the present study was to investigate the effect of cervical spine mobility alteration on the postural organisation of gait initiation in healthy young adults. Cervical spine mobility alteration was experimentally induced by the wearing of a cervical orthosis. APA, centre-of-mass braking, motor performance and/or postural stability were expected to be impaired under conditions of altered cervical spine mobility.

## Results

### Description of the biomechanical traces

**Figure 1:**
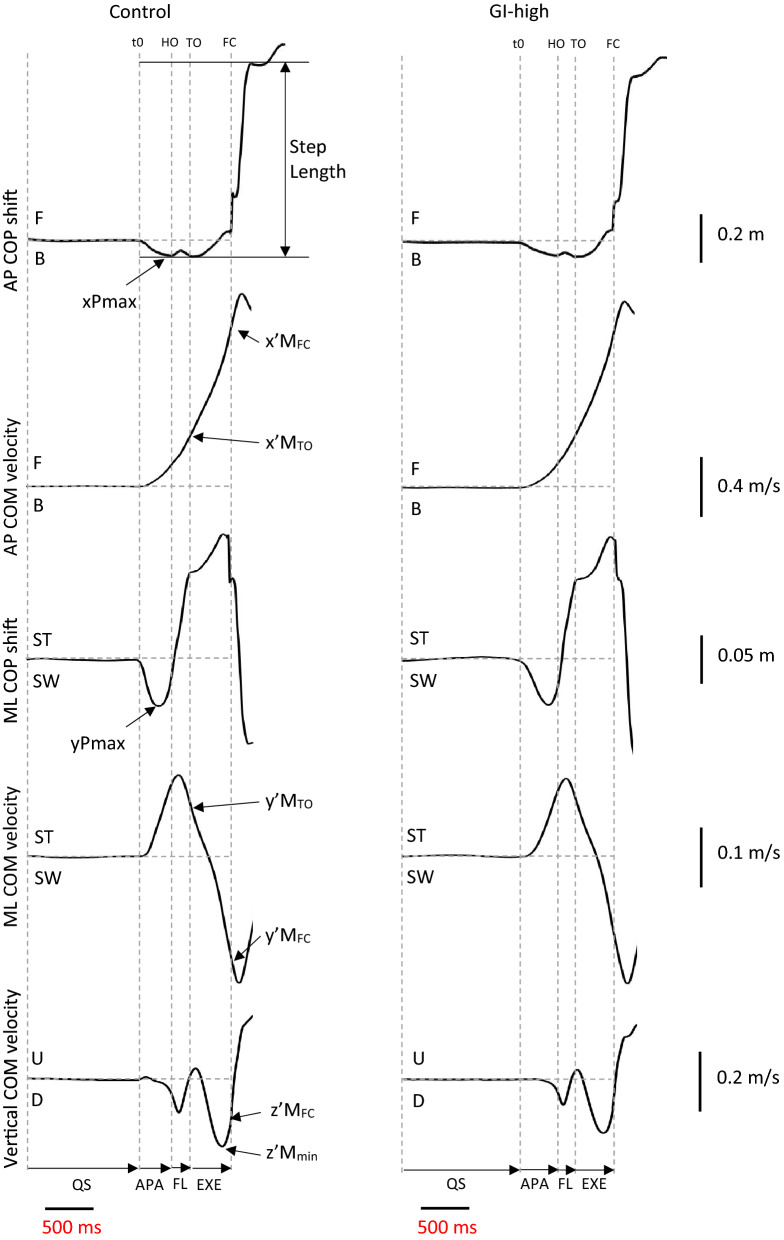
Typical biomechanical traces in the Control condition and the GI-high condition (gait initiation with a high level of cervical spine mobility restriction). Traces reported in each condition are obtained from the same participant performing one single trial. *Anteroposterior (AP) direction*. x’M_TO_, x’M_FC_: centre-of-mass (COM) velocity at toe-off and foot-contact. xPmax: peak of backward centre-of-pressure (COP) shift during APA. *F* forward; *B* backward. *Mediolateral (ML) direction*. y’M_TO_, y’M_FC_: COM velocity at toe-off and foot-contact. yPmax: peak of ML COP shift during APA. ST: stance limb; SW: swing limb. *Vertical direction*. z’M_min_, z’M_FC_: peak of negative vertical COM velocity and COM velocity at foot-contact. *D* downward; *U* upward. *Vertical dashed lines:* t0 onset variation of biomechanical traces; *HO* swing heel-off; *TO* swing toe-off, *FC* swing foot-contact. *Horizontal arrows*: *QS* quiet standing, *APA* anticipatory postural adjustments; FL: swing foot-lift; *EXE* execution phase of gait initiation.

In all four conditions, swing heel-off was systematically preceded by dynamic phenomena corresponding to APA. During these APA, the centre-of-pressure was shifted backward and toward the swing leg (Fig. 1). Mediolateral centre-of-mass velocity reached a peak value toward the stance leg at around swing toe-off, then the trace reversed direction to reach a peak value toward the swing leg a few milliseconds after foot-contact. Anteroposterior centre-of-mass velocity increased progressively to reach a forward-oriented peak a few milliseconds after foot-contact. The vertical centre-of-mass velocity reached a downward-oriented peak at around mid-stance. The trace then reversed direction, indicating that the centre-of-mass fall was actively braked during the execution phase.

### Initial posture, APA and foot-lift phase

**Figure 2:**
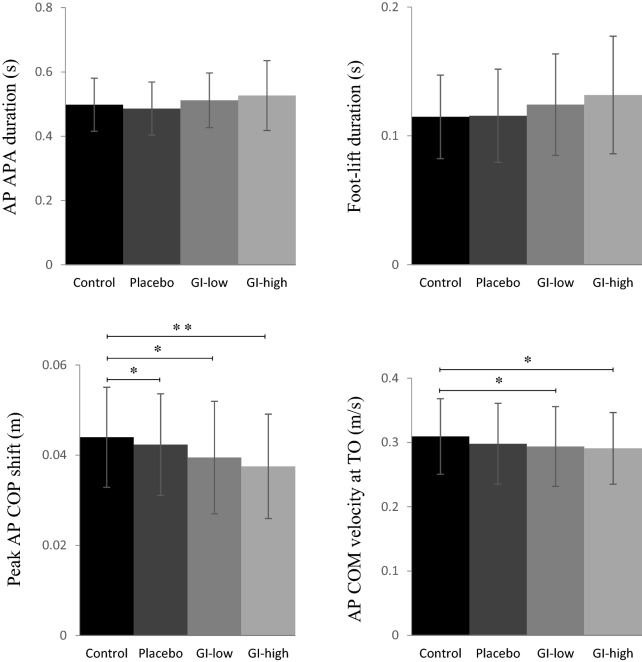
Effects of the condition on selected APA and foot-lift parameters. Reported are mean values (all participants together) + 1 SD. *APA* anticipatory postural adjustments, *AP* anteroposterior, *COP* centre-of-pressure, *COM* centre-of-mass, *TO* swing toe-off; *, **: significant difference between bars with *p* < 0.05 and *p* < 0.01, respectively.

There was no significant effect of the condition on the anteroposterior and mediolateral centre-of-mass position in the initial posture, or on any spatio-temporal parameters of APA along the mediolateral direction. By contrast, there was a significant effect of the condition on the peak of backward centre-of-pressure shift (F_3,42_ = 6.17, *p* < 0.01) and on forward centre-of-mass velocity at toe-off (F_3,42_ = 4.13, *p* < 0.05). Specifically, post hoc testing showed that the peak of backward centre-of-pressure shift was significantly higher in the control condition than in both the GI-high condition (*p* < 0.01; d = 0.57) and the GI-low condition (*p* < 0.05; d = 0.42). It was also significantly higher in the control condition than in the placebo condition (*p* < 0.05; d = 0.16). The forward centre-of-mass velocity was significantly higher in the control condition than in the GI-high condition (*p* < 0.05; d = 0.32). It was also higher in the control condition than in the GI-low condition (*p* < 0.05; d = 0.26). There was no significant effect of the condition on APA duration along the anteroposterior and mediolateral directions or on foot-lift phase duration (see Fig. 2).

### Motor performance

**Figure 3:**
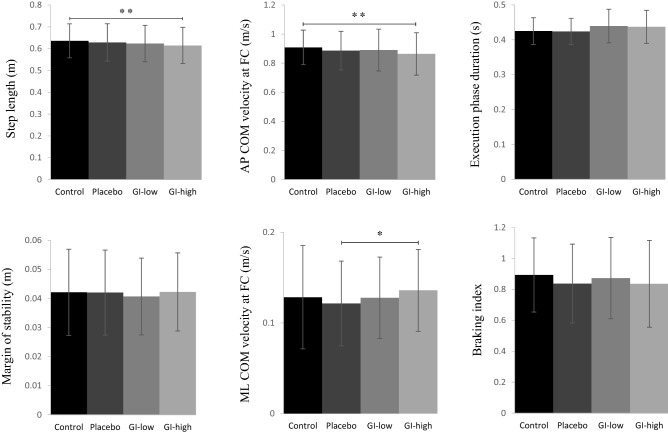
Effects of the condition on motor performance (upper panels) and stability parameters (bottom panels). Reported are mean values (all participants together) + 1 SD. *AP, ML* anteroposterior, mediolateral; *COM* centre-of-mass; *FC* foot-contact; *, **: significant difference between bars with *p* < 0.05 and *p* < 0.01, respectively.

There was a significant effect of the condition on step length (F_3,42_ = 5.07, *p* < 0.01) and on anteroposterior centre-of-mass velocity at foot-contact (F_3,42_ = 4.29, *p* < 0.01). Specifically, post hoc testing showed that both variables reached a significantly lower value in the GI-high condition than in the control condition (*p* < 0.01; d = 0.26 and d = 0.34, respectively). By contrast, there was no significant effect of the condition on the duration of the execution phase (Fig. 3).

### Stability

There was no significant effect of the condition on the margin of stability, base of support size and centre-of-mass shift at foot-contact. By contrast, there was a significant effect of the condition on mediolateral centre-of-mass velocity at foot-contact (F_3,42_ = 3.00, *p* < 0.05; Fig. 3). Post hoc analysis showed that this dependent variable was significantly higher in the GI-high condition than in the placebo condition (*p* < 0.05; d = 0.31).

### Vertical centre-of-mass braking

There was no significant effect of the condition on vertical centre-of-mass braking (*p* = 0.40) or on its components (Fig. 3), i.e. vertical centre-of-mass velocity at foot-contact (*p* = 0.19) or peak vertical centre-of-mass velocity reached during mid-stance (*p* = 0.24).

## Discussion

The aim of the present study was to investigate the influence of cervical spine mobility alteration induced by the wearing of orthoses with different degrees of rigidity (low or high), on the postural organisation of gait initiation in healthy young adults. APA, centre-of-mass braking, motor performance and/or postural stability were expected to be impaired under conditions of altered cervical spine mobility.

In line with these expectations, the results showed that the amplitude of APA along the anteroposterior direction, in terms of peak backward centre-of-pressure shift, decreased stepwise from the control condition to the GI-high condition. The difference between these two “extreme” conditions was 15% with a medium effect size (d = 0.57). This backward centre-of-pressure shift provides the initial (toe-off) forward centre-of-mass velocity necessary to reach the intended motor performance, in terms of forward progression velocity and step length^39^. Because the duration of APA along the anteroposterior direction remained unchanged across conditions, the results that both forward centre-of-mass velocity at toe-off and motor performance decreased with increasing cervical spine mobility alteration can be ascribed to this lower peak backward centre-of-pressure shift. Now, the difference in motor performance between the control condition and the GI-high condition was small, although significant (5% and 3% for forward centre-of-mass velocity at foot-contact and step length, respectively), as were the effect sizes (trivial and small, respectively).

These findings are partly in line with the study of Hamaoui and Alamini-Rodrigues on the effects of cervical spine mobility alteration on the postural and focal components of the sit-to-stand task. These authors reported that constraining cervical spine mobility by means of splints of different rigidity (low or high, as in the present study) resulted in an increased duration of the focal movement (corresponding to the delay between time of seat-off and time for stabilisation of the standing posture), and longer and larger APA, in terms of the backward centre-of-pressure shift. Based on these results, the authors suggested that restricted cervical spine mobility impaired the posturo-kinetic capacity of participants, leading to lower motor performance and a reorganisation of APA. This statement is in agreement with our results, although the findings reported by these authors contrast markedly with the results of the present study, where unchanged APA duration and decreased APA amplitude were found under conditions of restricted cervical spine mobility. In addition to the task difference between these two experiments, the discrepancy in the evolution of centre-of-pressure amplitude and APA duration with cervical spine mobility alteration could be ascribed to the different instructions on task velocity provided to participants by the experimenters: maximal velocity in Hamaoui and Alamini-Rodrigues, and spontaneous velocity in the present study. Sit-to-stand at a maximal velocity may have forced the postural system to develop adaptations of APA in the condition of restricted cervical spine mobility.

In contrast to the postural dynamics along the anteroposterior direction, both the APA along the mediolateral direction and the braking index remained unchanged across conditions. This finding suggests that these balance control mechanisms were not affected by cervical spine mobility alteration. It is noteworthy, however, that mediolateral centre-of-mass velocity at foot-contact, which is a component of the margin of stability, was significantly higher in the GI-high condition than in the placebo condition (the difference was 12%, with a small size effect). In other words, the velocity at which the centre-of-mass fell toward the swing leg side increased significantly with cervical spine mobility alteration, which has been thought to be a factor of reduced stability^24,40,41^. Previous studies showed that increasing whole-body stiffness during the execution phase of gait initiation resulted in lower postural stability, expressed by a lower margin of stability value^42^. In the present study, the increased centre-of-mass sideway velocity might be ascribed to a slight increase in whole-body stiffness (not measured) in the GI-high condition compared to the placebo condition. However, this negative effect would not be sufficient to impact postural stability significantly.

Cervical spine mobility alteration is known to be a cardinal symptom of PD. The results of the present study suggest that this symptom might contribute to the impaired APA associated with gait initiation^43–47^ or with upper limb tasks such as pointing^48^, and on the related motor performance classically observed in these patients. Similarly, Franzén et al. investigated the relationship between the magnitude of axial postural tone in the neck, trunk and hip segments in PD patients, and in several clinical functional tests (such as walking in a Figure of Eight, Timed Up & Go, Berg Balance Scale, Rollover test etc.). These authors showed that axial tone at these joint levels was related to functional performance, but most strongly for tone at the neck, and accounted for an especially large portion of the variability in the performance of the Figure of Eight test and the Rollover test. According to these authors, these results suggest that neck tone plays a significant role in functional mobility and that abnormally high postural tone may be an important contributor to balance and mobility disorders in individuals with PD. In the present study, neither the balance control mechanisms nor the margin of stability were affected by cervical spine mobility alteration. Now, it cannot be excluded that had a more complex task than gait initiation been tested, such as walking in a Figure of Eight test or the Rollover test, an alteration of these mechanisms might have been elicited. Future studies will investigate this hypothesis.

Because it contains the visual and the vestibular systems, the head needs to be stabilised in space to efficiently play its role as “inertial platform” from which postural control may be organised^32,34^. During locomotor activities such as gait initiation^17^, steady walking^35,49^, jumping^31–33^, etc., head-in-space stabilisation involves cervical spine mobility to efficiently compensate for the effects of trunk accelerations on head position^50^. Thus, under the conditions of restricted cervical spine mobility in the present study, the decrease in APA amplitude and related motor performance might reflect a cautious strategy directed to attenuate destabilisation of the head-in-space position, and facilitate postural control. This statement implies that cervical spine mobility would be a factor taken into account in the programming of APA associated with gait initiation. It follows that enhancing cervical spine mobility in patients with cervical spine rigidity, such as PD patients or the elderly, might be effective in improving the hypometrical APA generally observed in these populations^44,51,52^. Interestingly, Vialleron et al. recently showed that short-term stretching at the ankle joints (which are directly involved in the anticipatory backward^25,53^ and lateral centre-of-pressure shift^54^) was effective in improving APA and motor performance in PD patients^14^. Therefore, rehabilitation programs including cervical spine and ankle stretching may be beneficial in improving mobility at these joint levels and, hence, further enhance APA and motor performance in this population.

This study has at least two limitations. First, a priori sample size calculation was not conducted as the present study is not a clinical controlled randomised study. The sample size (n = 15 participants chosen randomly), however, falls within the classical range of papers in the literature focusing on the biomechanical organisation of gait initiation in healthy adult participants. Thanks to the coherence between the data (as stated throughout the discussion), we feel confident that this sample size is sufficient to reveal a consistent effect of cervical spine mobility restriction on gait initiation. Second, coordination between the head and trunk, as well as related head-in-space stability, were not quantified in the present study. However, since the function of the orthoses we used is to limit joint mobility, it can reasonably be speculated that these parameters were altered more greatly with increased orthosis rigidity.

In conclusion, the present study showed that the restriction of cervical spine mobility in healthy young participants altered the development of APA and, to a lesser extent, related motor performance, but left balance control mechanisms and stability unchanged. These effects might reflect the implementation of a more cautious strategy intended to attenuate head-in-space destabilisation due to trunk accelerations, and thus facilitate postural control. These results are in line with the posturo-kinetics capacity concept^2^, according to which APA and related motor performance depend on the mobility of the postural chain, i.e. cervical spine mobility in the present study. It follows that clinicians should be aware that the prescription of a rigid neck orthosis to patients with neurological conditions such as PD, or to elderly patients, could exacerbate pre-existing deficits in postural control during gait initiation.

## Methods

### Subjects

Fifteen healthy young adults (10 men and 5 women, aged 23.7 ± 1 years [mean ± 1 SD], height 1.7 ± 0.09 m, body mass 62.9 ± 16.6 kg), all free of any known neuromuscular disorder, participated in the present study. They gave written informed consent after being informed of the nature and purpose of the experiment, which was approved by the local ethics committee of the University Paris-Saclay. The study conformed to the standards set by the Declaration of Helsinki. The trial registration number was 2017-002814-30.

### Protocol

**Figure 4:**
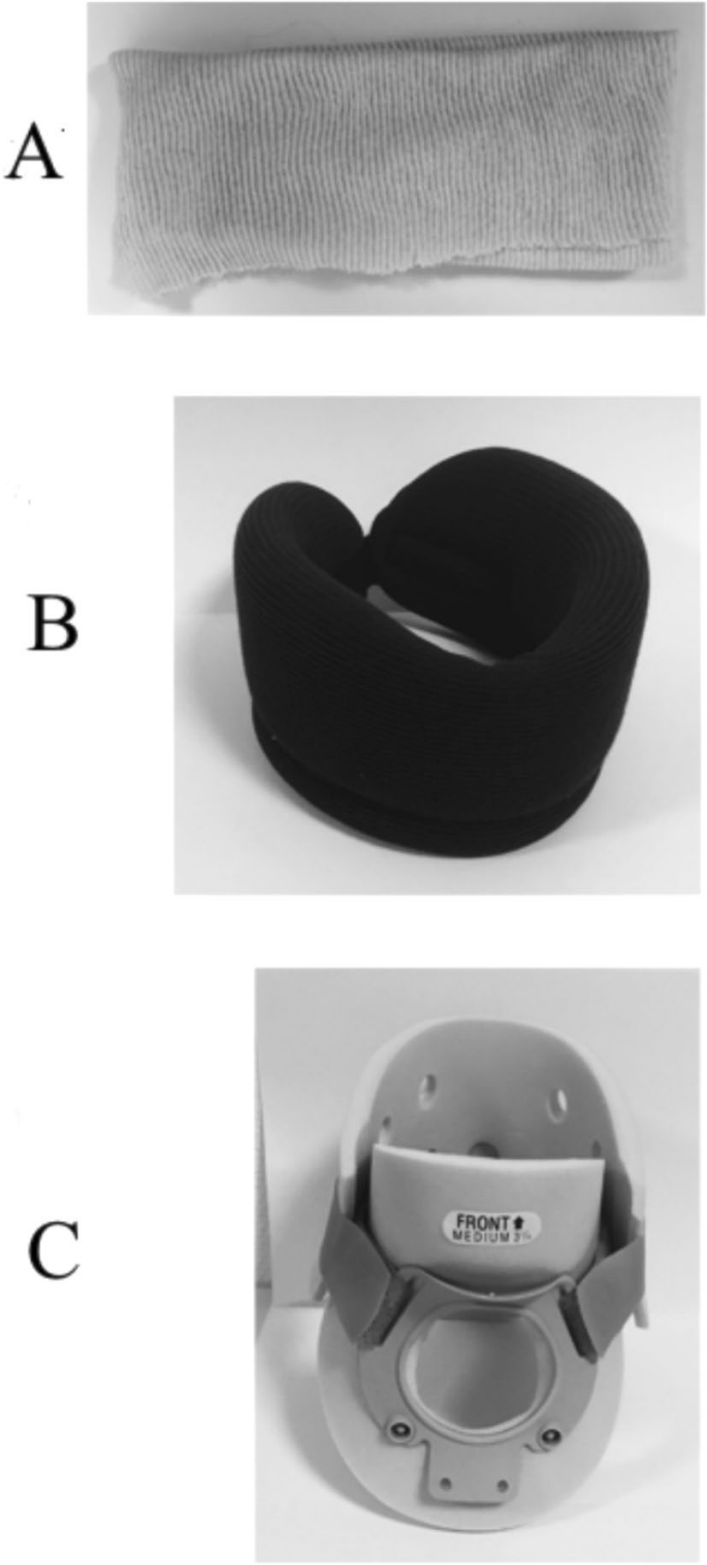
Cervical collars used in the placebo condition (**A**; jersey tubular bandage), the GI-low condition (**B**; foam flexible orthosis) and the GI-high condition (**C**; rigid orthosis).

The subjects stood barefoot in a natural upright posture on a force platform (0.9 × 1.80 m, AMTI, Watertown, USA) embedded at the beginning of a five-metre long track. From this initial posture, the subjects initiated gait at a self-determined speed following an acoustic signal, and continued walking straight to the end the track. The acoustic signal was delivered approximately five seconds after the start of data acquisition. It was made clear to the subjects that it was not a GO signal, and that they could start walking whenever they felt ready. The subjects performed series of ten trials in each of the following conditions: one control condition, one placebo condition and two test conditions. In the two test conditions, gait was initiated while wearing two different cervical orthoses that passively simulated low (GI-low condition, GI for “Gait Initiation”) and high levels of cervical spine mobility alteration (GI-high condition, Fig. 4^11,16,55^). A foam cervical orthosis (flexible collar 7.5 cm in height; Cooper, Melun, France) was used in the GI-low condition, and a Philadelphia orthosis (rigid collar 8.3 cm in height; Variteks, Istanbul, Turkey) was used in the GI-high condition. With the foam cervical orthosis, cervical flexion/extension is limited by 8–26%, lateral bending is limited by 8%, and rotation is limited by 10–17%^56^. The Philadelphia orthosis is a two-piece polyethylene foam collar that limits cervical flexion/extension by 59–75%, lateral bending by 12–34% and rotation by 27–56%^56^. In the placebo condition, a jersey tubular bandage (Neuss, Germany) was worn. This device does not limit neck mobility. It was used to test whether the changes in the sensory inputs from the neck skin receptors due to the wearing of a neck device could result in postural reorganisation of gait initiation. In the control condition, the subjects wore no neck device. These four conditions were assigned randomly across subjects.

A three-minute rest was imposed between two successive conditions to avoid the effects of fatigue. In each condition, the participants were allowed two familiarisation trials. Before the recordings, the experimenters determined the subjects’ preferential starting foot by pushing lightly against the subjects’ back while their eyes were closed to provoke a step forward^57^. The procedure was repeated three times. Then, the subjects were instructed to initiate gait with their “preferred” stepping leg systematically. The subjects were asked to repeat the trial when it was initiated with the “non-preferred” leg.

### Kinetics recordings and treatment

Data from the force platform were filtered using a no-lag low-pass Butterworth order with a 15 Hz cut-off frequency^58^. Instantaneous centre-of-mass accelerations along the anteroposterior, mediolateral and vertical directions were determined from the ground reaction forces according to Newton’s second law^23^. Centre-of-mass velocity and displacement were computed by means of numerical integrations of centre-of-mass acceleration using integration constants equal to zero, i.e. initial velocity was considered to be null^23^. The mediolateral (yCOP) and anteroposterior (xCOP) coordinates of the centre-of-pressure (COP) were computed from force platform data as follows:

1 $$yCOP=\frac{Mx+Fy\times dz}{Fz}$$

2 $$xCOP=\frac{-My+Fx\times dz}{Fz}$$

where Mx and My are the moments around the anteroposterior and mediolateral axes, respectively; Fx, Fy, and Fz are the anteroposterior, mediolateral and vertical ground reaction forces, respectively; and dz is the distance between the surface of the force-plate and its origin.

Data were collected at a rate of 500 Hz. Data acquisition was controlled by a custom-made program written in MATLAB [Version 5.3 (R11), The MathWorks Inc., United States].

### Experimental variables

#### Timing

The following instants were determined from the biomechanical traces: gait initiation onset (t0), swing heel-off, swing toe-off, swing foot-contact (Fig. 1). These instants were determined from force platform data^59,60^. Two t0 times were estimated, one for the mediolateral axis and one for the anteroposterior axis. The t0 times corresponded to the instants when the mediolateral or anteroposterior centre-of-pressure trace deviated 2.5 standard deviations from its baseline value.

#### Initial posture, APA and foot-lift phase

The mediolateral and anteroposterior centre-of-mass position in the initial upright static posture was estimated by averaging the centre-of-pressure position during the 250 ms period preceding the acoustic signal. Gait initiation was divided into APA (from t0 to swing heel-off), swing foot-lift (from swing heel-off to swing toe-off), and execution phase (from swing toe-off to swing foot-contact). The durations of APA along the mediolateral and anteroposterior axes were computed separately, because the t0 times for these two axes did not necessarily occur simultaneously^61^. The amplitudes of APA were characterised by the peaks of centre-of-pressure shift obtained during the APA time window and the centre-of-mass velocity/displacement along the mediolateral and anteroposterior directions at swing toe-off^62^.

#### Motor performance

Motor performance was evaluated with the following parameters: execution phase duration, anteroposterior centre-of-mass velocity at foot-contact and step length. Step length corresponded to the difference between the most backward centre-of-pressure position and the centre-of-pressure position at rear-foot clearance. This time was marked by the onset of the second plateau of the mediolateral centre-of-pressure trace^63^.

#### Vertical centre-of-mass braking

The “braking index” introduced by Do et al. (e.g.^64,65^) provides evidence that the centre-of-mass does not fall under the force of gravity but that the central nervous system prepares for foot-contact by decreasing the centre-of-mass vertical velocity to achieve a soft landing^26,27^. It was calculated as:

3 $$Braking\,Index=\frac{({z{^{\prime}}M}_{min}-{zt{^{\prime}}M}_{FC})}{{z{^{\prime}}M}_{min}}$$

where ${z{^{\prime}}M}_{min}$ is the minimal vertical centre-of-mass velocity and ${z{^{\prime}}M}_{FC}$ is the vertical centre-of-mass velocity at foot-contact^65^ (Fig. 1).

#### Stability

An adaptation of the “margin of stability” introduced by Hof et al. (2005) was used to quantify mediolateral dynamic stability at foot-contact^66^ (hereafter referred to as “Stability”). The margin of stability (MOS) corresponded to the difference between the mediolateral boundary of the base of support (BOS_ymax_) and the mediolateral position of the “extrapolated centre-of-mass” at swing foot-contact (YcoM_FC_). Thus:

4 $$MOS={BOS}_{ymax}-{YcoM}_{FC}$$

Based on the study by Hof et al. (2005) and the results of our previous studies (e.g.^42,61^), the mediolateral position of the extrapolated centre-of-mass at foot-contact was calculated as follows:

5 $$ YcoM_{{FC}}  = yM_{{FC}}  + \frac{{y^{\prime}M_{{FC}} }}{{\omega _{0} }} $$

where yM_FC_ and y’M_FC_ are respectively the mediolateral centre-of-mass position and velocity at foot-contact, and *ω*_0_ is the eigenfrequency of the body, modelled as an inverted pendulum and calculated as follows:

6 $${\omega }_{0}=\sqrt{\frac{g}{l}} $$

where *g* = 9.81 m/s^2^ is the gravitational acceleration and *l* is the length of the inverted pendulum, which in this study corresponded to 57.5% of body height^67^.

Stability at foot-contact is preserved on the condition that YcoM_FC_ is within BOS_ymax_, which corresponds to a positive margin of stability. A negative margin of stability indicates mediolateral instability and implies that a corrective action (e.g., in the form of an additional lateral step) is required to maintain balance.

### Statistics

Values of experimental variables were determined from the traces of each trial within one given condition and were then averaged across the ten trials of this condition. Thus, mean values and standard deviations were obtained for each variable in each condition. The Shapiro–Wilk test was used to check the normality of data distribution. A one-way repeated measures ANOVA with the condition (i.e. “control”, “placebo”, “GI-low”, “GI-high”) as within-subject factor was used. A significant outcome was followed by the Tukey post hoc test to assess statistical differences between pairs of conditions. The threshold of significance was set at *p* < 0.05. Cohen’s d was used to assess the effect sizes. The effect sizes were classified as trivial (< 0.2), small (0.2–0.49), medium (0.5–0.79) and large (≥ 0.8).

## Data Availability

The datasets generated during and/or analysed during the current study are available from the corresponding author on reasonable request.
